# Application of Micellar Extraction for Isolation of Famotidine from Aqueous Samples Prior to its Chromatographic Determination

**DOI:** 10.1007/s11743-017-2003-3

**Published:** 2017-08-12

**Authors:** Ilona Kiszkiel-Taudul, Barbara Starczewska, Joanna Karpińska, Monika Kasabuła

**Affiliations:** Institute of Chemistry, University of Bialystok, 15-245 Białystok, Poland

**Keywords:** Famotidine, SDS, Triton X-114, Micellar extraction, Surface water analysis, HPLC–UV

## Abstract

Micellar extraction was applied to isolate famotidine from aqueous samples. This drug is an H_2_ receptor antagonist used for the treatment of stomach diseases. The process was performed with a mixture of anionic sodium dodecylsulfate and nonionic Triton X-114 surfactants. The effect of different parameters on the efficiency of the micellar extraction such as electrolyte and surfactant concentration, pH of sample, temperature, shaking and centrifugation time was investigated. The influence of foreign substances on a studied process was tested. The elaborated procedure was applied for HPLC–UV determination of famotidine in natural water samples. The calibration graph was recorded in the range 1.35–37.12 μg mL^−1^ of the studied compound. The repeatability of the method was equal to 7.4%. The limit of detection and quantification values for the determination of famotidine by using the proposed method amounted to 0.40 and 1.25 μg mL^−1^, respectively.

## Introduction

Micellar extraction (ME) is successfully used for isolation of a variety of analytes from different matrices [1–4]. The ME process is based on the micellar aggregation of surface active agents (surfactants) and the phenomenon of cloud point and associated phase separation in nonionic surfactants. This isolation process is similar to liquid–liquid extraction, only an organic layer is generated in a homogeneous aqueous solution, which is converted to a heterogeneous phase. During micellar extraction, the analytes from the aqueous phase are solubilized in the hydrophobic core of the micelles [5, 6].

The level of pharmaceutical residues in environmental components is continually on the increase. This kind of contamination affects human and animal health [7]. Some of these pharmaceuticals are not biodegradable and are not eliminated during wastewater treatment [8, 9]. Pharmaceutical residues are detectable in surface-water samples in the order of magnitude of ng L^−1^ up to the μg L^−1^ [10]. Therefore, analysts are forced to develop new extraction methods for selective isolation and preconcentration of trace amounts of the studied compounds. Histamine H_2_ receptor antagonists belong to the compounds frequently found in raw sewage and wastewater treatment effluents, as well as in surface water, as a result of their common application. These drugs (namely, cimetidine, nizatidine, ranitidine and famotidine) are used for the treatment of diseases of the stomach. As famotidine (FMT) exhibits the highest activity in comparison to other compounds from this group, it is more frequently prescribed and as a consequence found in environmental samples [11, 12].

**Fig. 1:**
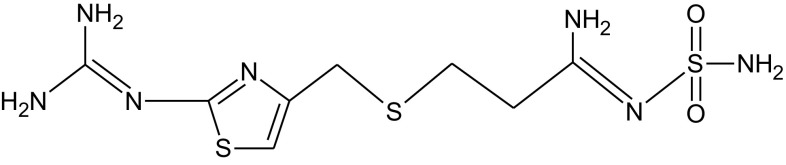
Chemical structure of famotidine

FMT (Fig. 1), [3-(((2-((aminoiminomethyl)amino)-4-thiazolyl)-methyl)thio)-N-(aminosulfonyl)propanimidamide], is applied in daily doses of 40 mg [13]. About 20% of FMT binds to plasma proteins and is metabolized to famotidine S-oxide. A large amount of unchanged drug is excreted with urine [14, 15]. FMT creates two polymorphic forms: aliphatic structure A (is more stable thermodynamically) and cyclic B [16, 17]. FMT exhibits basic properties (*pK*_a_ = 6.7) and is soluble in water and polar organic solvents [13]. This drug is able to form an ion-pair complex with acidic dyes and to coordinate transition metal ions due to the presence of thiazole nitrogen, thioether sulfur, guanidine and amine groups in its molecular structure [17–19].

To the best of our knowledge, micellar extraction has not been applied for the isolation of FMT. This compound was found in wastewater samples (in Taiwan, the western Balkan Region and Spain) at a very low levels, 0.008–0.094 μg L^−1^. Solid phase extraction with an Oasis HLB column was used for FMT isolation and preconcentration. Determination of FMT was performed by high-performance liquid chromatography coupled with mass spectrometry (HPLC–MS) [20] and tandem mass spectrometry (HPLC–MS/MS) [21–23]. LC–MS/MS method enabled the detection of FMT in the Danube river water samples (Hungary) at levels between 0.005 and 0.034 μg L^−1^ [24].

The present paper describes a newly elaborated micellar extraction procedure for the concentration of FMT from surface-water samples. The separation process was performed with a mixture of surface-active agents: anionic surfactant sodium dodecylsulfate (SDS) together with Triton X-114 (TX-114). This nonionic surfactant, with a cloud point of 25 °C, is representative of t-octylphenoxy polyoxethylene ethers [25, 26]. The determination of FMT was performed by spectrophotometric and HPLC methods with an ultraviolet detection system.

## Experimental

### Apparatus

Micellar extraction was performed using a vortex mixer (Heidolph Vibramax 110; Germany) and a centrifuge (MPW-251; Poland). Spectral measurements were carried out on a spectrophotometer UV/VIS (200 V, U-1900, model 3 IO-0003; Hitachi, Japan). Chromatographic analysis was performed using a HLPC system (Thermo Separation, USA) including a detector Spectra System UV 3000 equipped with a low gradient binary pump P2000 and a Rheodyne injector with 20-μL sample loop.

### Reagents and Solutions

All solutions were prepared using double-distilled water. The active substance, FMT, was obtained from Sigma-Aldrich (USA). A standard solution of FMT (1687.40 μg mL^−1^) was prepared by dissolving 0.169 g in 100 mL of water with the addition of a drop of concentrated hydrochloric acid.

The surfactants, SDS and TX-114, were obtained from Sigma-Aldrich. A standard solution of SDS (0.2 mol L^−1^) was prepared by dissolving 5.768 g in a 100-mL volumetric flask with water. A stock solution of TX-114 (5% w/v) was prepared by diluting 100% (w/v) solution of TX-114 in a 100-mL flask.

The electrolytes, sodium chloride, calcium chloride, potassium bromide, and sodium sulfate, were obtained from Poch (Gliwice, Poland). Standard solutions of the chlorides (4 mol L^−1^), bromide (4 mol L^−1^) and sulfate (1.5 mol L^−1^) were prepared by dissolving appropriate amounts in 100 mL of water.

Methanol, acetonitrile, dipotassium hydrogen phosphate, sodium hydroxide, and hydrochloric and phosphoric acids were obtained from Poch. A stock solution of hydrochloric acid and sodium hydroxide (0.1 mol L^−1^) was prepared by dissolving appropriate amounts in a 1000 mL volumetric flask. Working solutions of hydrochloric acid and sodium hydroxide (10^−2^ mol L^−1^) were prepared by diluting the stock solutions (0.1 mol L^−1^) in 100 mL of water.

### Micellar Extraction Procedure

For the micellar extraction of FMT, 1 mL of an aqueous solution of SDS (0.2 mol L^−1^) and 0.16 mL of 5% solution of Triton X-114 were transferred into a 10 mL volumetric tube. Then 0.1 mL of 1687.40 μg mL^−1^ FMT standard solution and 2 mL of 4 mol L^−1^ NaCl solution were added and the mixture was diluted to 10 mL with doubly distilled water. The content of the tube was shaken for 15 min. The separation of the two phases was achieved by centrifugation operated for 10 min at 5800 rpm. The formation of micelles was observed as the white sediment in the volumetric tube.

After removing the water phase, the surfactant-rich layer in the tube was dissolved and diluted to 10 mL with methanol. The final concentration of FMT in micellar phase was calculated to be 16.87 μg mL^−1^. The absorbance of extracts was measured at 275 nm.

### Analysis of Aqueous Samples

Surface water samples were collected from a local river (hospital region, Podlaskie Voivodeship in Poland). Aqueous samples were taken according to analytical sampling requirements on about 1/3 river depth about 50 cm from the river bank into polyethylene flasks. Before conducting experiments, samples of water were filtered through the paper filters to remove solid particles. The micellar extraction (procedure 2.3) was used for the determination of FMT in natural aqueous samples. The developed isolation method allows preconcentration of FMT. The final volume of micellar extracts dissolved in methanol was equal to 2 mL. Afterwards, the analysis of river water was performed by applying the elaborated extraction process and preconcentration of real samples.

### Chromatographic Analysis (HPLC–UV)

The chromatographic separation was performed on a Lichrospher ^®^ 100 RP-18 column (125 mm × 4.6 mm, 5 μm) using as a mobile phase, a mixture of acetonitrile/50 mmol L^−1^ potassium dihydrogen phosphate (60:40 v/v), adjusted to pH of value 3.5 by concentrated phosphoric acid. A flow rate of 1 mL min^−1^ was maintained while the wavelength was set at 275 nm. A retention time of 1.01 min was applied for the determination of FMT.

## Results and Discussion

### Primary Studies

FMT exhibits basic properties and it exists in a cationic form in aqueous solutions in the pH range: 4–7. Therefore, an anionic surface active agent, sodium dodecylsulfate (SDS), was used for solubilization of this drug [27]. It was supposed that the isolation process of the analyte into surfactant-rich phase is a result of electrostatic interaction between organic cation of FMT and dodecylsulfate anion, which form ion pair [FMT]^+^ [SDS]^−^. FMT exists in neutral and anionic forms in basic aqueous solutions.

**Fig. 2:**
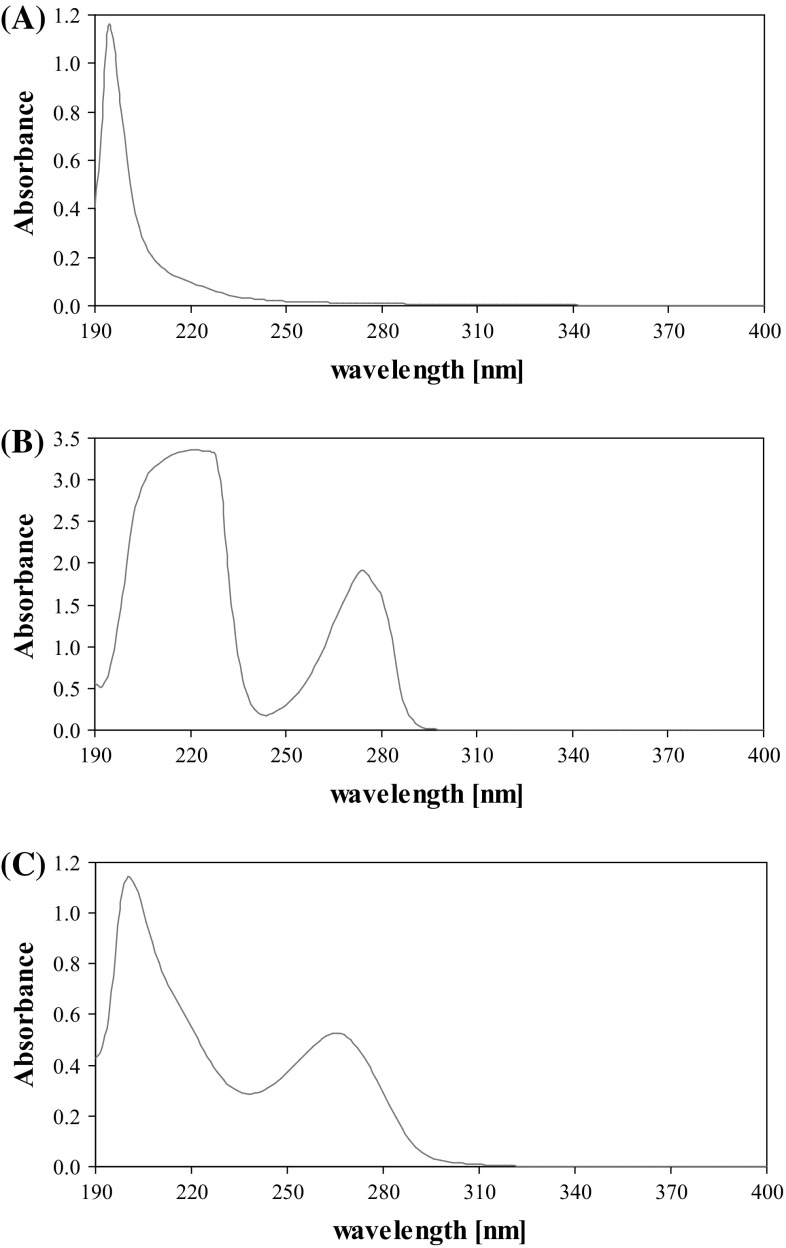
Absorption spectra of FMT and solutions of surfactants in methanol before micellar extraction: SDS, 0.02 mol L^−1^ (**a**); TX-114, 0.08% (**b**); FMT, 16.87 μg mL^−1^ (**c**)

**Fig. 3:**
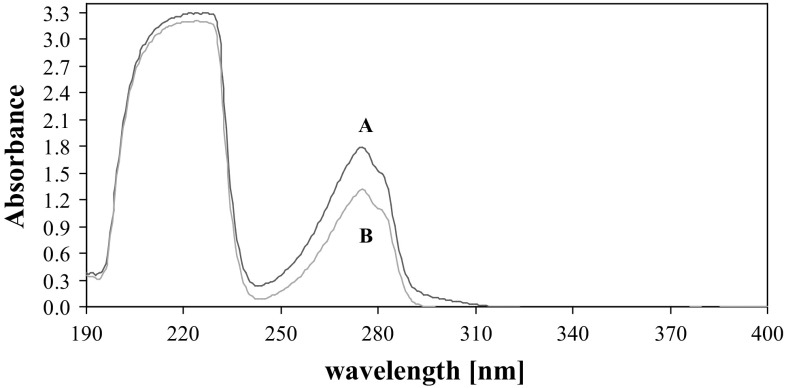
Absorption spectrum of micellar extract of FMT (16.87 μg mL^−1^) in methanol (**a**) and blank solution (**b**) after micellar extraction. Conditions: SDS, 0.02 mol L^−1^; TX-114, 0.08%; NaCl, 0.8 mol L^−1^

The primary studies showed that the used surfactant was able to bind FMT in micelles. It was observed that the addition of the non-ionic surface active agent: Triton X-114 enhanced the efficiency of micellar extraction. Absorption spectra of applied solutions of surfactants and the studied analyte (FMT) are shown in Fig. 2 while the spectra of micellar extract of FMT and a blank solution of used surfactants without the analyte are presented in Fig. 3. A micellar extract of FMT exhibits an intense band at 275 nm. This wavelength was subsequently used for the measurements of absorbance in optimization of the best parameters for the micellar extraction.

### Selection of Surfactants

The effect of surfactant concentration on the efficiency of the process of FMT isolation was investigated. The extraction was performed using a mixture of surfactants (SDS and TX-114) above their critical micellar concentration, which is 8.2 × 10^−3^ mol L^−1^ for SDS and 0.27 × 10^−3^ mol L^−1^ for TX-114 [28, 29].

For this purpose, different amounts of TX-114 were added into the 10 mL volumetric tubes at the fixed concentration of SDS (0.02 mol L^−1^), FMT (1687.40 μg mL^−1^) and sodium chloride (0.6 mol L^−1^). In the second series of samples, various volumes of SDS at the fixed amounts of TX-114 (0.08%), drug and electrolyte were transferred into the volumetric tubes. The samples were shaken and centrifuged for 10 min. The absorbance of the surfactant-rich phases was measured at 275 nm. Absorption spectra of methanolic extracts were registered against the blank solution.

**Fig. 4:**
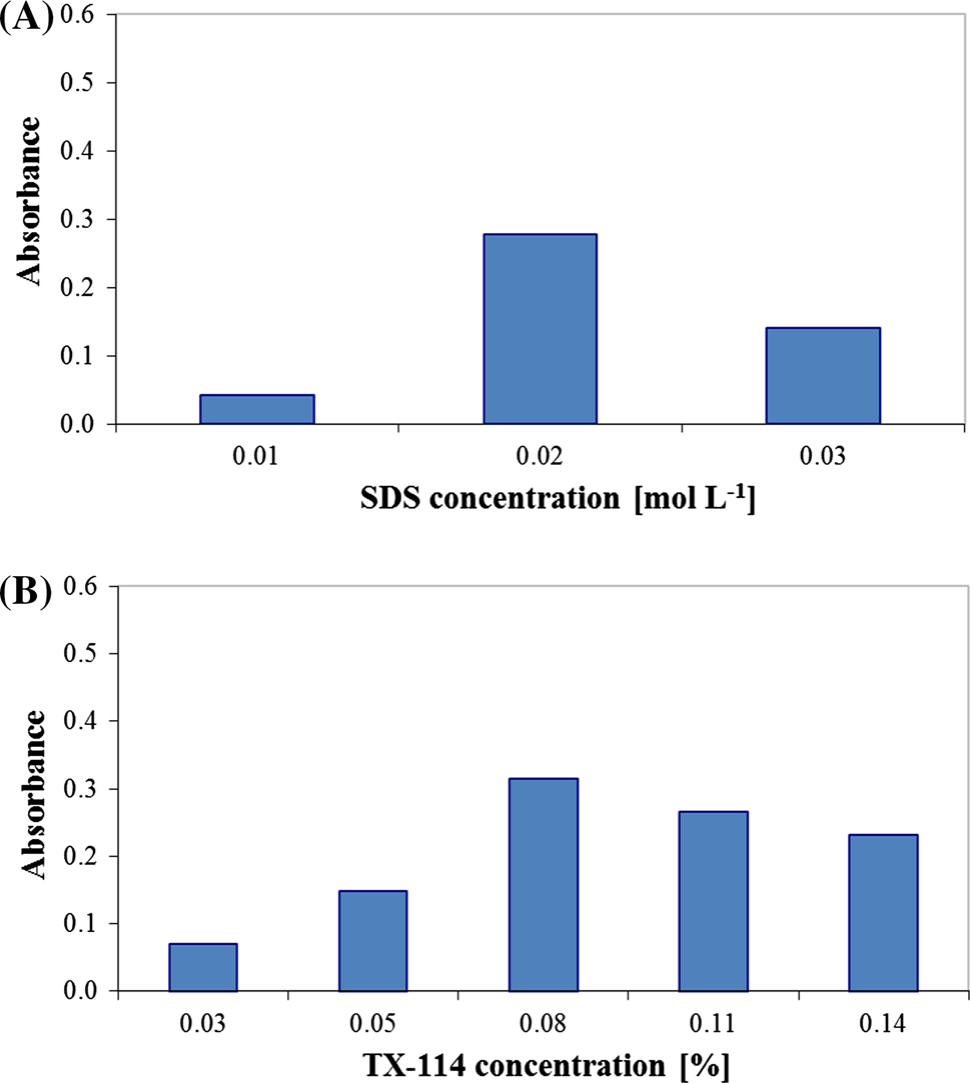
The influence of surfactant concentration on the absorbance of FMT

The best efficiency of micellar extraction of FMT was obtained using 0.02 mol L^−1^ SDS and 0.08% TX-114 (Fig. 4). It was observed that the use of other values of concentration of surfactants during isolation of FMT, caused a decrease in the efficiency of micellar extraction of the studied analyte.

### Effect of Electrolyte

It was observed that an addition of an electrolyte influenced the isolation process of the studied compound. The addition of salt increases the density of the aqueous layer and enhances the separation of the two phases.

The effect of the following electrolytes: potassium bromide (KBr), sodium sulfate (Na_2_SO_4_), calcium chloride (CaCl_2_) and sodium chloride (NaCl) on the efficiency of the extraction of FMT was investigated. The micellar extraction of a series of samples containing the mixture of surfactants (0.02 mol L^−1^ SDS and 0.08% TX-114), FMT (1687.40 μg mL^−1^) and the electrolytes was performed. The final concentration of added salts in the micellar phase was calculated to be 0.6 mol L^−1^.

**Fig. 5:**
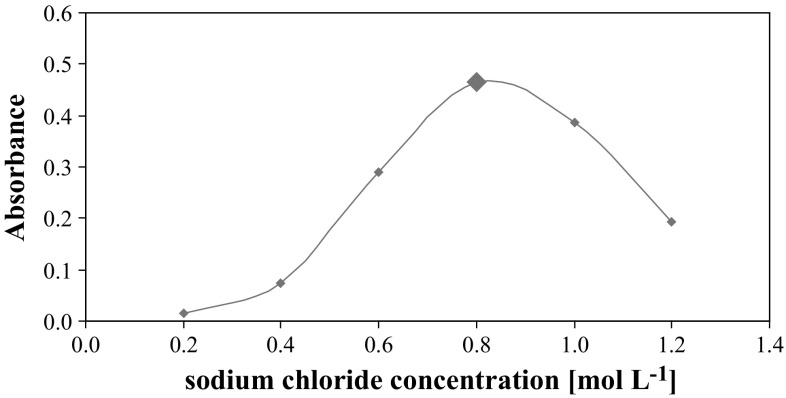
The effect of sodium chloride concentration on the absorbance of FMT using a mixture of surfactants (SDS and TX-114). The *large data point* indicates the condition chosen during optimization of micellar extraction of FMT

It was found that the addition of sodium chloride into FMT solution improved the formation of micelles and increased the efficiency of the extraction of FMT. The concentration of NaCl was optimised. The extraction was performed with the use of the fixed concentration of surfactants and FMT while varying the concentration of sodium chloride in the range 0.2–1.2 mol L^−1^. The obtained results (Fig. 5) showed that the absorbance of the micellar extracts increased with an increase in the NaCl concentration up to 0.8 mol L^−1^. Above this value, the absorbance of extracts decreased and the efficiency of the micellar extraction of FMT was lowered. Therefore, 0.8 mol L^−1^ of sodium chloride was chosen and used in the subsequent investigations.

### Choice of pH Sample

The influence of the concentration of hydrogen ions on the efficiency of the micellar extraction of FMT was investigated. The initial pH of the prepared mixture of reagents was 6.45.

The process of FMT isolation was performed using the optimized concentration of surfactants (0.02 mol L^−1^ SDS, 0.08% TX-114) and the electrolyte (0.8 mol L^−1^). Each desired value of pH was adjusted by the addition of a suitable volume of hydrochloric acid (0.1 and 0.01 mol L^−1^) or sodium hydroxide (0.1 and 0.01 mol L^−1^). The influence of pH on FMT micellar extraction process was studied in the range 2.20–10.64.

**Fig. 6:**
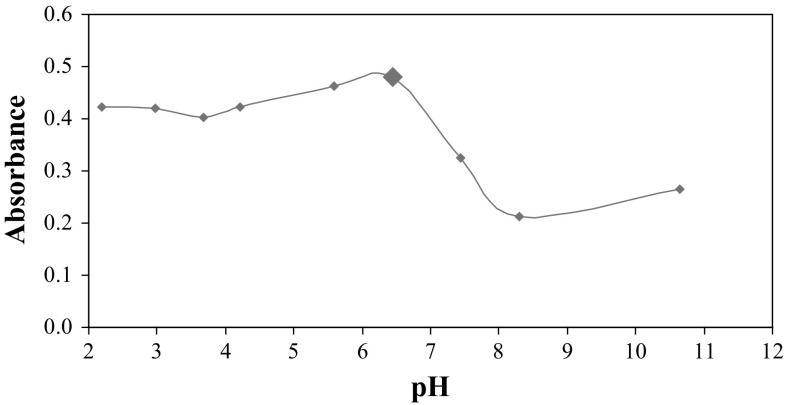
The influence of the pH of the prepared samples on the absorbance of FMT. The *large data point* indicates the condition chosen during optimization of micellar extraction of FMT

Figure 6 shows the dependence of absorbance of FMT extracts on the pH of the solution. It was observed that absorption values of the received extracts are stable for the studied samples in the pH range 2.20–6.45. Above this value, the efficiency of FMT micellar extraction dramatically decreased. The obtained results led to the conclusion that changing the pH of the solution of reagents is not necessary. Thus, micellar extraction of FMT at pH 6.45 was performed for further studies.

### Effect of Shaking and Centrifugation Times and Temperature

The influence of shaking and centrifugation times on the micellar extraction of FMT was studied. The isolation process of the analyte was performed using the optimized concentrations of the surfactants and the electrolyte, applying variable shaking and centrifugation times during the micellar extraction. It was observed that 15 min of shaking and 10 min of centrifugation of the samples is adequate for the micellar extraction of FMT. The application of a time shorter than the optimal value caused difficulty in mixing the sample components and separating the phases. A longer time of shaking (more than 15 min) and the centrifugation (more than 10 min) resulted in a decrease in the efficiency of the isolation process of FMT.

The effect of temperature on the micellar extraction of FMT in the range of 40–80 °C was studied. It was found that this parameter had no influence on the isolation process. Therefore, 25 °C (cloud point of TX-114) was chosen for FMT extraction [30].

### Effect of Foreign Ions on the Efficiency of Micellar Extraction of FMT

The influence of interfering ions commonly present in surface-water samples on the isolation of FMT was studied.

The micellar extraction of FMT (1687.40 μg mL^−1^) with the addition of variable amounts of foreign species was performed. The prepared samples were shaken for 15 min, then they were centrifuged for 10 min. The absorbance of micellar layers (dissolved in methanol) was measured at 275 nm. The tolerance limit was defined at the level of the interferents causing an error of ±5% in FMT concentration. The obtained results are presented in Table 1. It was observed that the proposed extraction procedure was tolerant of the ions commonly present in surface waters.

**Table 1:** Tolerance limits of foreign ions for determination of 16.87 μg mL^−1^ FMT after micellar extraction

Interferents	Excess of foreign ions in ratio to content of FMT in the sample
PO_4_^3−^	300
NO_3_^−^	140
NH_4_^+^	110
Mn^2+^	300
Ca^2+^	300
CO_3_^2−^	300
Cl^−^	500
Br^−^	500
SO_3_^2−^	400
SO_4_^2−^	400

The examined organic substances, cysteine, glucose, lactose, chloramine, citric acid, tartaric acid and other biologically active compounds, such as olanzapine, cimetidine, ranitidine, carbamazepine and lovastatin, do not influence the results of the determination of the studied analyte.

### Chromatographic Analysis

The developed micellar extraction procedure was applied for sample preparation prior to chromatographic determination of FMT. The selection of an appropriate mobile phase was carried out first. For this purpose, various mixtures of acetonitrile:50 mmol L^−1^ potassium dihydrogen phosphate; acetonitrile:doubly distilled water; acetonitrile:methanol; acetonitrile:acetate buffer; and methanol:50 mmol L^−1^ potassium dihydrogen phosphate were investigated at different volumetric ratios..

**Fig. 7:**
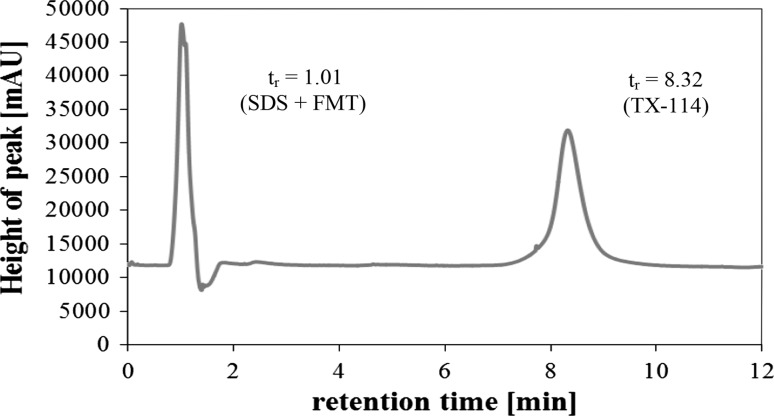
Chromatogram of micellar extracts of FMT. Conditions: FMT, 16.87 μg mL^−1^; SDS, 0.02 mol L^−1^; TX-114, 0.08%; NaCl, 0.8 mol L^−1^

It was observed that the studied mobile phases did not cause the separation of FMT and SDS peaks. The use of the mixture of acetonitrile:50 mmol L^−1^ potassium dihydrogen phosphate (60:40 v/v, pH = 3.5) as an eluent allowed a satisfactory separation of the surfactants in spite of the fact that FMT and SDS eluted together. Chromatographic peaks of FMT and SDS appeared at 1.01 min while TX-114 appeared at 8.32 min (Fig. 7). It was observed that the SDS peak in a chromatogram of a blank extract was remarkably smaller than this in the chromatogram of the sample. Therefore, chromatographic analysis was performed on a Lichrospher^®^ 100 RP-18 column using as a mobile phase the mixture of CH_3_CN/50 mmol L^−1^ KH_2_PO_4_ (60:40 v/v, pH = 3.5). The flow rate was 1 mL min^−1^. The quantitation of FMT was carried out by calculating the difference between the peak of the areas of a blank solution and samples under the same conditions.

### Calibration and Validation

Next using the optimized conditions, a calibration graph was recorded. For this purpose, a series of FMT solutions at the concentrations 1.35, 3.37, 6.75, 16.87, 30.37, and 37.12 μg mL^−1^ were prepared and subjected to the micellar extraction. The chromatograms of the extracts were recorded. The calibration graph was drawn as a function of the difference between the sample peak area and the blank peak against the concentration.

The precision of the chromatographic method for FMT determination followed by the micellar extraction was estimated. Reproducibility was determined by recording calibration curves for a few days with a varied FMT concentration in the range 1.35–37.12 μg mL^−1^. In order to determine repeatability, extraction under optimal conditions was performed for five samples containing the studied analyte (16.87 μg mL^−1^). The limit of detection (LOD) and quantification (LOQ) of the method for FMT determination was calculated as the minimum detectable amount of FMT with a signal-to-noise ratios of 3 and 10, respectively.

The coefficient of preconcentration for extraction of FMT was determined to enable the detection of FMT in real samples. For this purpose, the calibration graph with preconcentration of the analyte was recorded under the experimental conditions. Micellar extracts were dissolved in 2 mL of methanol. This is the minimal amount of organic solvent which was needed to dissolve micelles after extraction. The preconcentration factor was calculated as the ratio of the slope of the calibration graph to that without the preconcentration of FMT. The analytical parameters are shown in Table 2. Chromatographic determination of FMT with the micellar extraction is characterized by a large range of linearity. The obtained results are reproducible. The low values of limit detection and quantification enable the detection of FMT in surface-water samples at μg mL^−1^ level. The coefficient of the preconcentration for the micellar extraction of FMT was equal to 6.7.

**Table 2:** Analytical characteristics ofthe chromatographic method for the determination of FMT with an application of micellar extraction

Analytical parameter	Micellar extraction of FMT—HPLC–UV
Equation of calibration curve (*n* = 5)	*y* = 2.3 × 10^9^x + 1.4 × 10^4^
Slope ± standard deviation (SD)	2.3 × 10^9^ ± 3.2 × 10^8^
Intercept ± SD	1.4 × 10^4^ ± 1.0 × 10^4^
Coefficient of correlation ± SD	*R* ^2^ = 0.9979 ± 0.006
Linearity	1.35–37.12 μg mL^−1^
Repeatability (*n* = 5)	7.4%
Reproducibility	14.0%
Limit of detection (LOD) (the minimum detectable amount of analyte with a signal-to-noise ratio of 3)	0.40 μg mL^−1^
Limit of quantification (LOQ) (the minimum detectable amount of analyte with a signal-to-noise ratio of 10)	1.25 μg mL^−1^
Coefficient of preconcentration	6.7

The developed procedure of micellar extraction of FMT using a mixture of surfactants is characterized by a larger efficiency of the isolation process and a higher value of the coefficient of the preconcentration in comparison to the procedure of the extraction of FMT with the application of a single surfactant (SDS) [27]. It was observed that the developed method features a larger range of linearity (1.35–37.12 μg mL^−1^) and a lower LOD (0.40 μg mL^−1^) in comparison to the extractional–spectrophotometric method described by Abu et al. [18]. The proposed liquid–liquid extraction based on complexation reaction with bromocresol green enables the spectrophotometric determination of FMT in the range of the concentration, 2.0–23.6 μg mL^−1^. The LOD value of this method is equal to 2.0 μg mL^−1^. The advantage of the elaborated micellar extraction is a decrease in the amounts of toxic and flammable organic solvents in comparison to a traditional liquid–liquid extraction. The developed micellar extraction procedure for the chromatographic determination of FMT is characterized by a lower value limit of quantification, 1.25 μg mL^−1^, in comparison to LOQ, 3.0 μg mL^−1^, for the chromatographic (HPLC–UV) method of detection of FMT reported by Ashiru et al. [31].

### Chromatographic Determination of FMT in River Water Samples

The proposed micellar extraction procedure using a mixture of the surfactants was applied to the determination of FMT in surface water. The samples were obtained from the local rivers (Podlaskie Voivodeship, Poland).

The micellar extraction of surface water samples was performed using the optimal conditions (Procedure 2.3.). In the first series of experiments, the river samples were spiked with standard FMT (3.37 μg mL^−1^). Then, a micellar process for the isolation of FMT was performed for real samples, which contained an analyte at the concentration of 16.87 μg mL^−1^. The pH of the samples was adjusted to 6.45.

Methanolic extracts were subjected to the HPLC–UV analysis and the results are presented in Table 3. The obtained values of the extraction recovery (about 100%) indicated the absence of FMT in the real samples in the studied range of concentration. The micellar extraction for preconcentration of the river samples without the addition of an analyte was performed to confirm the obtained results. It was observed that the peak area of SDS at the retention time 1.01 min in a chromatogram of methanolic extracts was the same as in the case of the standard solution. These results confirmed the non-detection of FMT in the studied surface-water samples.

**Tabel 3:** Results of FMT in river samples by the proposed chromatographic method with isolation by micellar extraction

Surface-water samples	Micellar extraction of FMT; determination method: HPLC–UV			
	Added amount (μg)	Found amount ± SD (μg) (*n* = 3)	Average recovery ± SD (%) (*n* = 3)	RSD (%)
River Horodnianka	33.75	33.86 ± 1.36	100.3 ± 4.0	4.0
	168.74	171.38 ± 3.10	101.6 ± 1.8	1.8
River Dzierzbia	33.75	33.52 ± 2.58	99.3 ± 7.6	7.7
	168.74	168.85 ± 5.38	100.1 ± 3.2	3.2
River Narew	84.37	83.92 ± 1.36	99.5 ± 1.6	1.6
River Netta	101.24	101.81 ± 3.60	100.6 ± 3.6	3.5

### Conclusions

A new isolation procedure for FMT from an aqueous solution is proposed. The most important advantage of the developed procedure in comparison to a classical liquid–liquid extraction is a significant decrease in the amount of the used organic solvent, and therefore micellar extraction is relatively friendly to the environment. During the performance of this isolation process, the analytes are not lost by evaporation of solvents and are not adsorbed to glass surfaces. The extraction method with the use of surfactants is characterized by cost-effectiveness and is easily applied for the separation of different kinds of analytes (for example, drugs, proteins, metal ions) from complicated matrices. The application of micellar extraction for FMT using a mixture of surfactants (sodium dodecylsulfate and Triton X-114) enables the isolation of the studied analyte with high efficiency and suitable selectivity. The developed method for the isolation and determination of FMT allows an enrichment of the analyte, and it can be used for the analysis of environmental samples, pharmaceutical preparations and biological fluids. The average value of a recovery for various concentrations of FMT was equal to 104.2 ± 9.9%. A combination of micellar extraction of FMT with its chromatographic determination enables the assaying of FMT in a wide range of linearity. The value of LOD shows the possibility of the determination of the drug in studied samples at the level of μg mL^−1^. The obtained results indicated that FMT was not detected in the water samples in the studied range of concentration.
